# Bacteriophage P2-71: a promising therapeutic against multidrug-resistant *Proteus mirabilis* in urinary tract infections

**DOI:** 10.3389/fvets.2024.1445264

**Published:** 2024-09-17

**Authors:** Ruihu Wu, Zhiyou Dong, Yunjiang Liu, Jialiang Xin, Yuxi Duan, Haohong Zheng, Yizhou Yang, Hualin Fu, Zhijun Zhong, Haifeng Liu, Ziyao Zhou, Yixin Huang, Guangneng Peng

**Affiliations:** Key Laboratory of Animal Disease and Human Health of Sichuan Province, College of Veterinary Medicine, Sichuan Agricultural University, Chengdu, China

**Keywords:** bacteriophage, alternative therapy, *Proteus mirabilis*, multidrug-resistance, biofilm, treatment

## Abstract

**Background:**

*Proteus mirabilis* is a Gram-negative, rod-shaped bacterium widely found in natural environments. It is known for causing a range of severe illnesses in mammals, particularly urinary tract infections (UTIs). This study evaluates the therapeutic efficacy of phage P2-71 against *Proteus mirabilis in vivo* and *in vitro* environments.

**Methods:**

The *in vitro* therapeutic potential of bacteriophage P2-71 was assessed through the ability of phage to kill *Proteus mirabilis* by using a plate counting assay, and biofilm inhibition and biofilm lysis assays using a microtitre plate method. Additionally, an *in vivo* UTI model in C57BL/6Jmice was developed via urethral inoculation of the bacterium. Phage therapy was administered through urethral injection over a period of 5 days. Therapeutic outcomes were measured by analyzing bacterial load, phage titer, inflammatory markers, and histopathological changes in the urine, urogenital tissues, and spleen.

**Results:**

*In vitro*, bacteriophage P2-71 achieved significant reductions in *P. mirabilis* concentrations, with log reductions of 1.537 and 0.7009 CFU/mL in laboratory and urine environments, respectively (*p* < 0.001). The phage also decreased biofilm formation by 34–49% and lysed 15–25% of mature biofilms at various multiplicities of infection (MOIs) (*p* < 0.001). *In vivo*, phage treatment significantly lowered bacterial concentrations in the urine on Days 1 and 3 (*p* < 0.0001), achieving a maximum reduction of 4.602 log₁₀ CFU/mL; however, its effectiveness diminished by Day 5 (*p* > 0.05). Concurrently, phage titers decreased over time. Importantly, phage treatment notably reduced bacterial load in the bladder, kidneys, and spleen (*p* < 0.001). Inflammatory markers such as IL-6, IL-1β, and TNF-*α* were significantly lower in the treatment group, especially in the bladder (*p* < 0.0001), indicating an effective reduction in inflammation. Histopathological analysis showed significant mitigation of tissue damage.

**Conclusion:**

The results demonstrated that bacteriophage P2-71 is a promising alternative therapy for UTIs caused by MDR *Proteus mirabilis*. This bacteriophage therapy offers a viable strategy for managing infections where traditional antimicrobials fail, highlighting its potential in clinical applications.

## Introduction

*Proteus mirabilis* is a zoonotic bacterium that is ubiquitously present in the natural environment. Clinically, it is known to form crystalline biofilms on both the external surface and internal cavity of urethral catheters due to its ureolytic biomineralization capabilities (1). This biofilm formation often leads to catheter encrustation and blockage, commonly resulting in urine retention and ascending urinary tract infections (UTIs), which can cause cystitis, pyelonephritis, bladder or kidney stones, and in severe cases, potentially fatal complications such as septicemia and endotoxic shock (2).

In human medicine, *P. mirabilis* is a predominant pathogen in catheter-associated UTIs, attributed to its robust biofilm-forming and urease-producing abilities (3–5). Recent studies have indicated an increasing prevalence of *P. mirabilis* infections in companion animals, with isolation rates as high as 30% in regions such as northeast China and Chengdu (6–12). The overuse of antibiotics in veterinary medicine has exacerbated this situation, leading to a rise in multidrug-resistance (MDR) and extensively drug-resistant strains of *P. mirabilis* (13–16). Moreover, there is evidence to suggest that UTIs in companion animals and humans may be caused by closely related strains of *P. mirabilis*, sharing similar antibiotic resistance genes (ARGs), thus posing a potential public health risk (17).

Given these challenges, there is a critical need to explore alternative treatments that can effectively combat MDR *P. mirabilis*. Bacteriophages, or phages, are viruses that infect specific bacterial hosts and are considered promising candidates for this purpose. Phages replicate within their host cells and are expelled from the body when the host is absent, providing a self-limiting treatment mechanism. They offer several advantages, including the ability to proliferate at the site of infection, specificity toward the target bacteria without affecting the commensal flora, and efficacy against biofilms and other structures that contribute to antibiotic resistance (18–20). Phages have demonstrated effectiveness against various bacterial infections in animals, including those caused by *Staphylococcus aureus*, *Cronobacter*, *Acinetobacter baumannii*, and *Escherichia coli* (21–26). More importantly, a large amount of research data has shown that the use of single phages or phage cocktails in the treatment of urinary tract infections is effective with negligible safety concerns, suggesting that phage therapy is a successful alternative or supplemental to antibiotic therapy (27).

In a previous study, we successfully isolated and biologically characterized phage P2-71. Phage P2-71 exhibits a very short latency and a strong burst size and remains stable in the temperature range of 30°C-50°C and at a pH of 4–11. Moreover, phage P2-71 was found to be lethal to *P. mirabilis* 37 at different MOI levels by the analysis of killing curves. We also revealed the genetic composition of phage P2-71 by genomic analysis, including genes necessary for morphogenesis, lysis and DNA modification, and did not find resistance and virulence genes. This demonstrated its potential against MDR *P. mirabilis* (28). The aim of this study was to further analyze the efficacy of phage P2-71 in the treatment of UTIs caused by MDR *P. mirabilis* through comprehensive *in vivo* and *in vitro* evaluations.

## Materials and methods

### Experimental bacterial strains

This study utilized a multidrug-resistant *P. mirabilis* strain, designated *P. mirabilis* 37, which was isolated from canine feces and preserved in our laboratory’s repository. Serving as the host for phage P2-71, *P. mirabilis* 37 was characterized for its multidrug resistance (MDR) profile. According to the criteria established by the Clinical and Laboratory Standards Institute (CLSI), multidrug-resistance was confirmed based on its resistance to at least three different classes of antimicrobial agents. Specifically, susceptibility testing revealed that *P. mirabilis* 37 exhibited resistance to Cefazolin, Imipenem, Meropenem, and Tetracycline (9).

### Propagation and purification of phage P2-71

Propagation began by mixing a logarithmic-phase culture of *P. mirabilis* 37 with bacteriophage P2-71 at a multiplicity of infection (MOI) of 0.1, to which 5 mL of LB medium was added. This mixture was incubated overnight at 37°C with constant shaking at 220 rpm. Following incubation, the mixture was centrifuged at 8,000 × g for 10 min at 4°C, and the supernatant was retained. The supernatant then underwent ultracentrifugation at 30,000 rpm for 4 h at 4°C. The resulting pellet was resuspended and subjected to sucrose density gradient centrifugation under the same conditions for an additional 4 h, enabling the isolation of the phage band. This isolated phage layer was subsequently diluted by half and subjected to low-speed centrifugation at 8,000 × g for 10 min at 4°C to remove excess sucrose. This sucrose removal process was repeated three times. Finally, the concentrated phage P2-71 was stored at a refrigeration temperature of 4°C (28).

### Efficacy of phage P2-71 against planktonic *Proteus mirabilis* 37 in PBS buffer

The bactericidal efficacy of phage P2-71 against planktonic cells of *P. mirabilis* 37 was assessed in a PBS environment, using a methodology adapted from (29). 100 μL of an overnight culture of *P. mirabilis* 37, with a bacterial concentration of 10^8^ CFU/mL, was mixed with 100 μL of phage P2-71 at a concentration of 10^9^ PFU/mL (MOI = 10). This mixture was then added to 10 mL of PBS buffer and incubated in a bacterial incubator at 37°C. Bacterial concentrations were measured at eight time points: 0, 2, 4, 6, 8, 10, 12, and 24 h post-infection, using gradient dilutions plated on *Salmonella*-*Shigella* (SS) medium.

### Efficacy of phage P2-71 against planktonic *Proteus mirabilis* 37 in artificial urine

To evaluate the bactericidal efficacy of phage P2-71 against planktonic cells of *P. mirabilis* 37 in a urinary environment, the method previously described was employed. Specifically, 100 μL of an overnight culture of *P. mirabilis* 37 at a concentration of 10^8^ CFU/mL was mixed with 100 μL of phage P2-71 at a concentration of 10^9^ PFU/mL (MOI = 10). This mixture was then added to 10 mL of artificial urine (Phygene, Fuzhou, China) and incubated in a bacterial incubator at 37°C. Bacterial concentrations were measured by collecting samples at time intervals of 0, 2, 4, 6, 8, 10, 12, and 24 h post-infection. Gradient dilutions of these samples were spread on Salmonella-Shigella (SS) medium to assess the bacterial counts.

### Biofilm growth cycle of *Proteus mirabilis* 37

*Proteus mirabilis* 37 was incubated overnight at 37°C to initiate growth. After this, the bacterial concentration was measured and diluted to 10^7^ CFU/mL in broth medium. One hundred microliters of this bacterial suspension was then added to 96-well cell culture plates in three replicates and incubated at 37°C. Samples were removed at 12, 24, and 36 h to assess biofilm development. Following each incubation period, the culture solution was discarded, and the plates were washed three times with phosphate-buffered saline (PBS) and air-dried. Adherent bacteria were fixed in each well with 200 μL of 98% methanol for 10 min, after which the methanol was removed, and the plates were left to air-dry. The biofilms were stained with 1% crystal violet for 45 min, eluted with 33% acetic acid, and the optical density at 590 nm was measured using a microplate reader to quantify biofilm formation.

### Evaluation of the efficacy of phage P2-71 in the prevention of biofilm formation

The efficacy of phage P2-71 in preventing biofilm formation by *P. mirabilis* 37 was assessed following a 24-h co-culture period, using a methodology adapted from Maszewska’ study (30). Initially, *P. mirabilis* 37 was incubated overnight at 37°C. Post-incubation, the bacterial concentration was determined and adjusted to 10^7^ CFU/mL. Subsequently, 100 μL of this bacterial suspension was dispensed into each well of a 96-well cell culture plate. To each well, 100 μL of phage P2-71, prepared at various MOIs of 100, 10, 1, 0.1, and 0.01, was added. The plates were then incubated at 37°C for 24 h. Following incubation, the extent of biofilm formation was quantified using the same method described previously. Control groups included a negative control (phage only) and a positive control (bacteria only).

### Estimation of phage P2-71 effectiveness in biofilm eradication

To assess the capability of phage P2-71 to disrupt mature biofilms, we employed a modified version of the method described by Maszewska’ study (30). *P. mirabilis* 37 was cultured overnight and diluted to a concentration of 10^7^ CFU/mL. This bacterial suspension was then transferred to a 96-well cell culture plate and incubated at 37°C for 24 h. After incubation, the culture solution was discarded, and the wells were gently rinsed three times with 100 μL of PBS to remove non-adherent cells. Subsequently, 100 μL of phage P2-71, prepared at various MOIs of 100, 10, 1, 0.1, and 0.01, was added to each well. The plates were then returned to the incubator and maintained at 37°C for an additional 24 h before biofilm quantification.

For biofilm assessment, the wells were stained using crystal violet, and the biofilm mass was quantified using a spectrophotometer. To validate the results, control groups were included: a negative control containing only the phage solution and a positive control containing only the bacterial culture.

### *In vivo* phage therapy using a mouse model

In this study, we evaluated the efficacy of phage therapy using a modified mouse model, adapted from (31). Female C57BL/6 J mice, 7 weeks old, were acclimatized for 1 week with unrestricted access to food and water. The *P. mirabilis* 37 was prepared by centrifuging 50 μL of bacterial culture containing 10^8^CFU, discarding the supernatant, and resuspending in phosphate-buffered saline; this process was repeated twice. Concurrently, a phage P2-71 solution was prepared at 10^9^PFU in a 50 μL volume and kept at 4°C for stability. Under isoflurane anesthesia, a 28G catheter was inserted into the bladder via the urinary tract, and 50 μL of the prepared bacterial solution was injected, retained for 1 min to prevent backflow. The physiological state of the mice was monitored throughout the procedure, with warmth maintained post-anesthesia until recovery (animal testing approval number: 2022303145).

The experiment was divided into 4 groups: a positive control injected with *P. mirabilis* 37, a negative control receiving only phage, a mock control given PBS, and the treatment group was treated with a urethral injection of 50 μL of phage P2-71 containing 10^9^ PFU 6 h after bacterial challenge, followed by phage injection every 24 h until the fifth day. Each group consisted of 12 mice.

### Assessment of bacterial load, phage titer, cytokine levels, and histopathological changes

*In vivo* monitoring of bacterial load and phage titer was conducted by collecting urine samples from the mice on the Day 1(6 h after phage treatment), Day 3, and Day 5 post-treatment. On the fifth day, following the final urine collection, mice were euthanized, and their bladder, kidney, and spleen tissues were harvested and homogenized in 1 mL of PBS. These homogenates were then subjected to gradient dilution and analyzed using SS medium and the double plate method to determine the bacterial load and phage titer.

Concurrently, cytokine assays were performed on the same tissues. After homogenization, the samples were centrifuged at 10,000xg for 10 min at 4°C, and the supernatants were collected. The concentrations of interleukins IL-6, IL-1β, and TNF-*α* in these supernatants were quantified using ELISA kits (Kexing Trading, Shanghai, China).

Histopathological changes were assessed through light microscopic examination of tissue sections. Following the protocol outlined by (25), dissected tissues were fixed in 10% neutral buffered formalin, embedded in paraffin, sectioned at 5 microns, and stained with hematoxylin and eosin. The stained sections were then analyzed for morphological alterations.

### Statistical analysis

Statistical analysis was performed using t test followed by Tukey’s multiple comparison statistical test, GraphPad Prism® Software version 9.5.0. A probability value (*p*-value) <0.05 was considered significant.

## Result

### Susceptibility of planktonic cells of *Proteus mirabilis* 37 to phage P2-71 in PBS buffer

**Figure 1:**
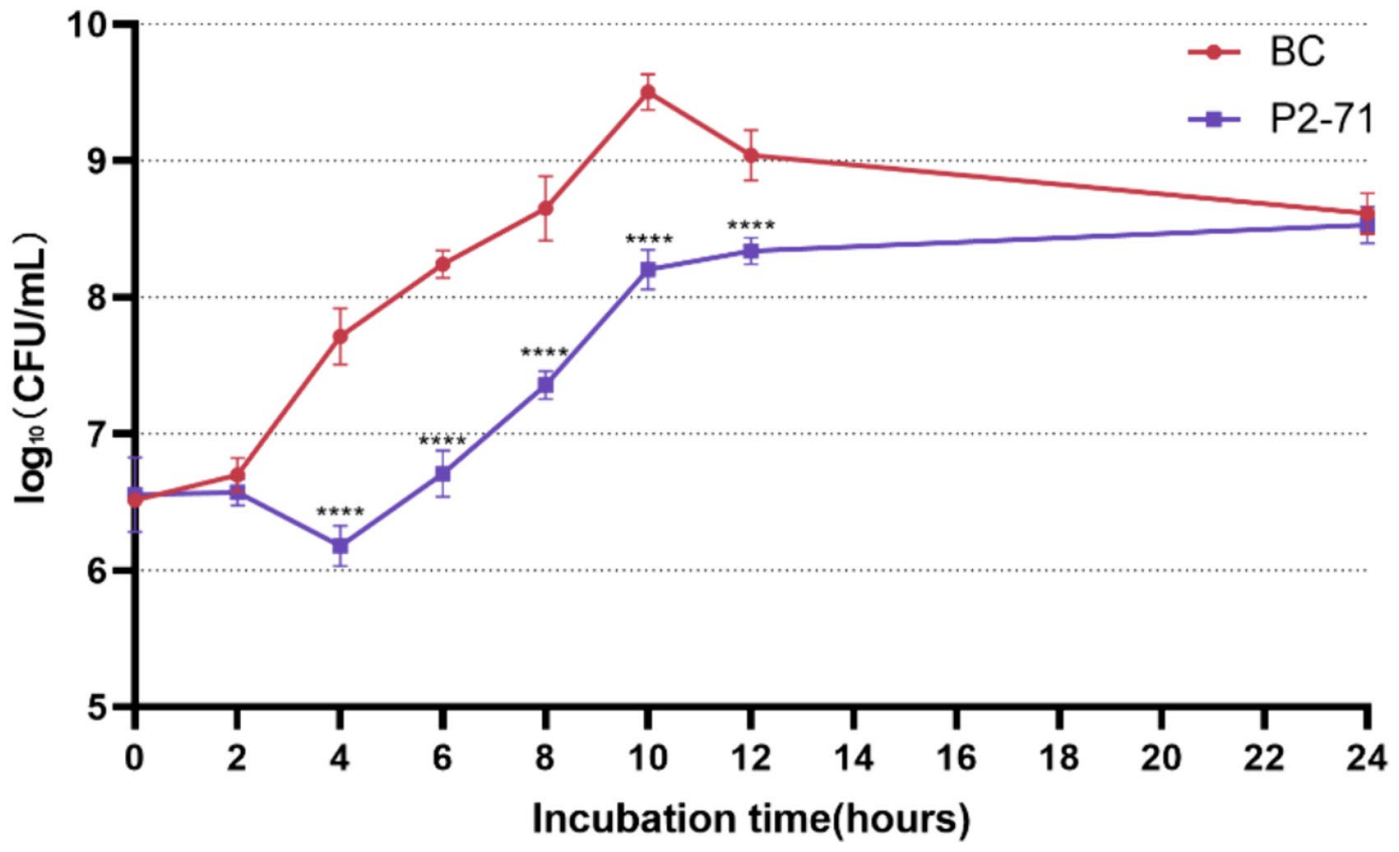
Efficacy of Phage P2-71 Against Planktonic *P. mirabilis* 37 in PBS Buffer. BC represents the control group containing only *P. mirabilis* 37, and P2-71 represents the phage treatment group. Values represent the mean of three independent experiments, and error bars indicate standard deviation. Statistical analysis was performed using *t* test followed by Tukey’s multiple comparison statistical test, ****, *p* < 0.0001.

The efficacy of phage P2-71 in reducing the concentration of planktonic *P. mirabilis* 37 cells in PBS was quantitatively assessed over a 24-h period. The data demonstrate a significant reduction in bacterial counts from 4 to 12 h post-infection, with reductions of 1.535, 1.537, 1.293, 1.299, and 0.6988 log₁₀ CFU/mL at each two-hour interval, respectively (*p* < 0.0001). Notably, the phage’s bactericidal activity was not evident during the initial 2-h period (*p* > 0.05), nor was it significant at the 24-h mark (*p* > 0.05), indicating a temporal limitation in the phage’s effectiveness (Figure 1).

### Susceptibility of planktonic cells of *Proteus mirabilis* 37 to phage P2-71 in an artificial urine environment

**Figure 2:**
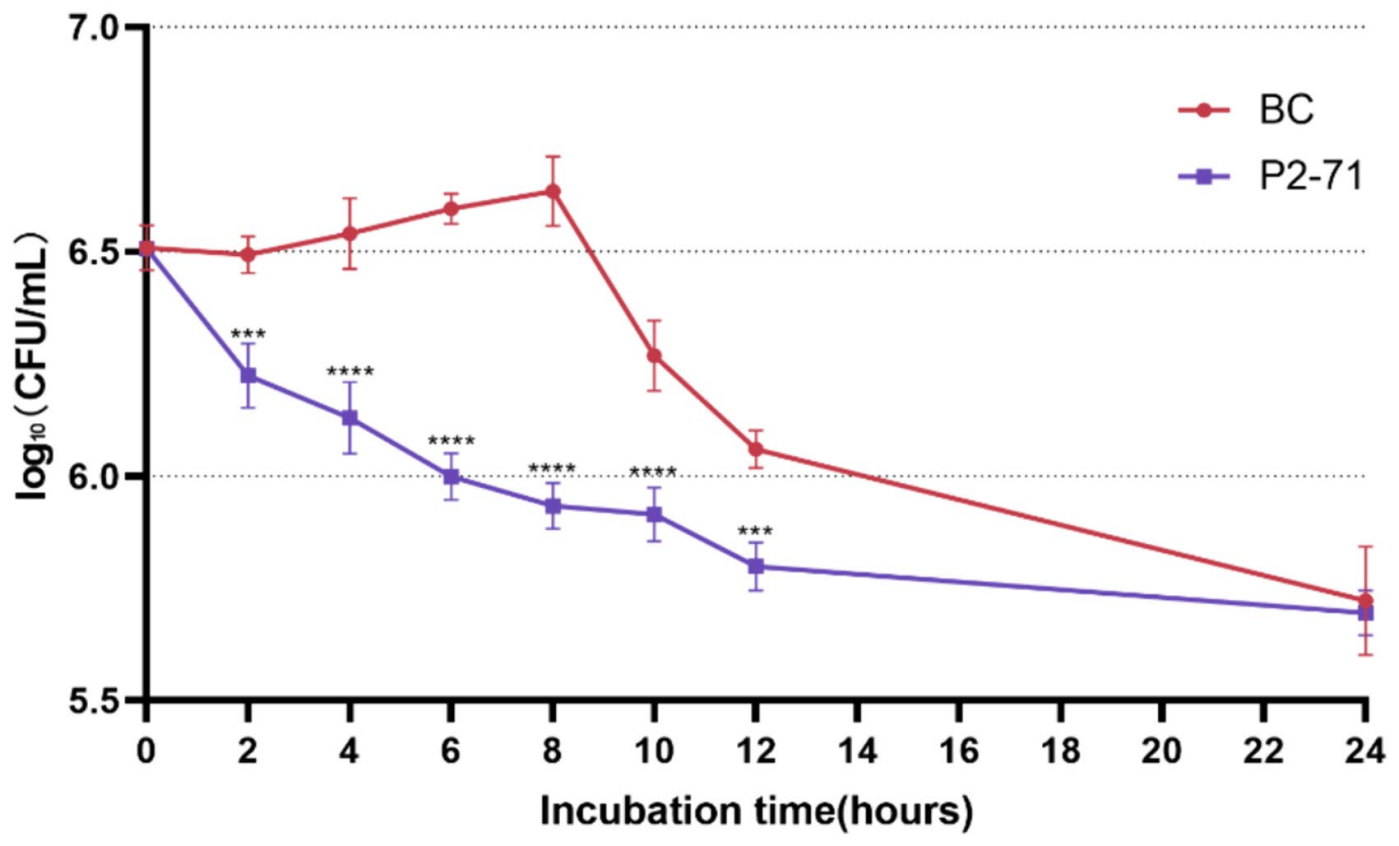
Efficacy of Phage P2-71 Against Planktonic *P. mirabilis* 37 in Artificial Urine. BC represents the control group containing only *P. mirabilis*, and P2-71 represents the phage treatment group. Values represent the mean of three independent experiments, and error bars indicate standard deviation. Statistical analysis was performed using t test followed by Tukey’s multiple comparison statistical test, ***, *p* < 0.001, ****, *p* < 0.0001.

Phage P2-71 demonstrated significant bactericidal activity against *P. mirabilis* 37 in a simulated urinary environment. Notably, the phage effectively reduced bacterial counts between 2 and 12 h post-infection, with log₁₀ reductions of 0.2639, 0.4109, 0.5961, 0.7009, 0.3530, and 0.2613 CFU/mL at each subsequent two-hour interval. The most pronounced reduction occurred at 8 h (*p* < 0.001). However, by 24 h, the phage’s effect diminished, and no significant reduction in bacterial load was observed (*p* > 0.05; Figure 2).

### Biofilm growth cycle of *Proteus mirabilis* 37

**Figure 3:**
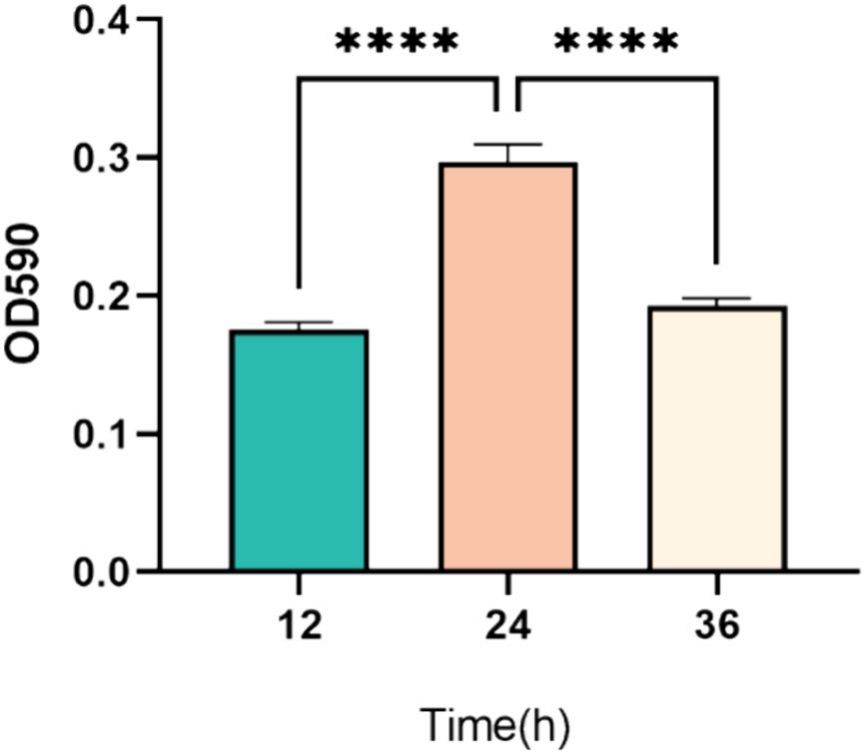
Temporal dynamics of biofilm formation by *P. mirabilis* 37 over a 36-h period. The graph represents the average optical density at 590 nm (OD_590_) from three independent experiments, indicating biofilm growth. Error bars denote the standard deviation of the means. Statistical significance was determined using a One-Way ANOVA followed by Tukey’s multiple comparisons test, where **** indicates a *p*-value of <0.0001.

As illustrated in Figure 3, the optical density measurements indicate significant changes in biofilm formation over time. Specifically, biofilm formation at 24 h was significantly higher than at both 12 h and 36 h (*p* < 0.001). This suggests that biofilm formation by *P. mirabilis* 37 was still in the early stages at 12 h, reached its peak or maturation at 24 h, and began to enter the detachment phase by 36 h (Figure 3).

### Evaluation of the efficacy of phage P2-71 in the prevention of biofilm formation

**Figure 4:**
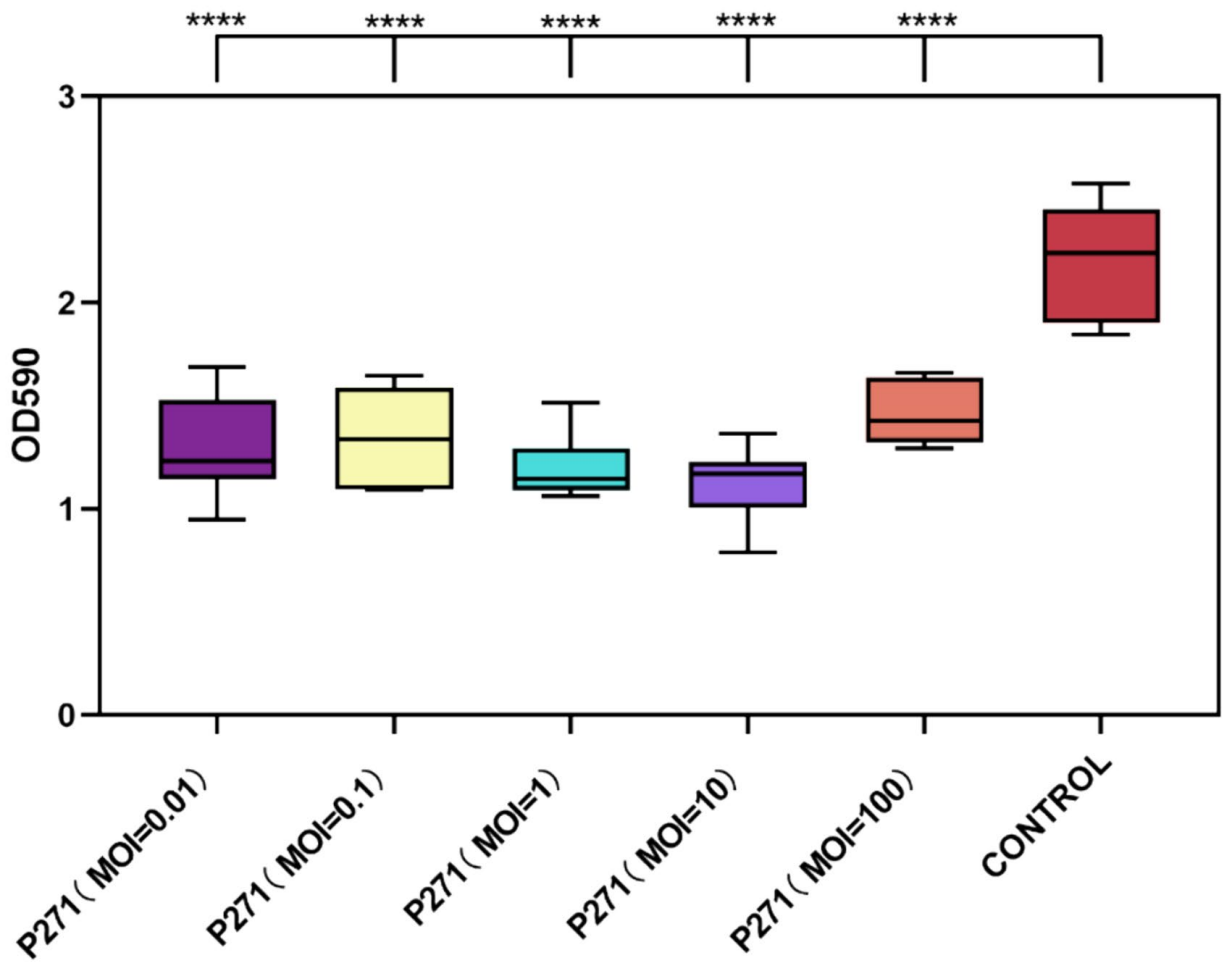
Inhibitory effect of phage P2-71 on biofilm formation by *P. mirabilis* 37 at various MOIs over 24 h. The control represents biofilm levels of untreated *P. mirabilis* 37, whereas the phage-treated groups illustrate the biofilm density following treatment with phage P2-71 at different MOIs. Data are presented as the mean optical density at 590 nm (OD_590_) from three independent experiments. Statistical significance was assessed using One-Way ANOVA followed by Tukey’s multiple comparisons test, where **** indicates a *p*-value of <0.0001.

A Phage P2-71 exhibited significant efficacy in preventing biofilm formation by *P. mirabilis* 37 when co-cultured for 24 h at various MOIs. Biofilm formation was reduced by 34 to 49%, demonstrating the phage’s potential as a preventive agent against biofilm development. Specifically, reductions were 41% at MOI = 0.01, 39% at MOI = 0.1, 46% at MOI = 1, 49% at MOI = 10, and 34% at MOI = 100. These results indicate a consistent and significant decrease in biofilm density across all tested MOIs compared to the control, with each group showing highly significant reductions (*p* < 0.001), as illustrated in Figure 4.

Despite the variability in reduction percentages, statistical analysis revealed no significant differences between the MOIs themselves in their capacity to inhibit biofilm formation (*p* > 0.05). This suggests that even at the lowest MOI tested, phage P2-71 is effective at inhibiting biofilm development, supporting its use across a range of concentrations without loss of efficacy.

### Estimation of phage P2-71 effectiveness in biofilm eradication

**Figure 5:**
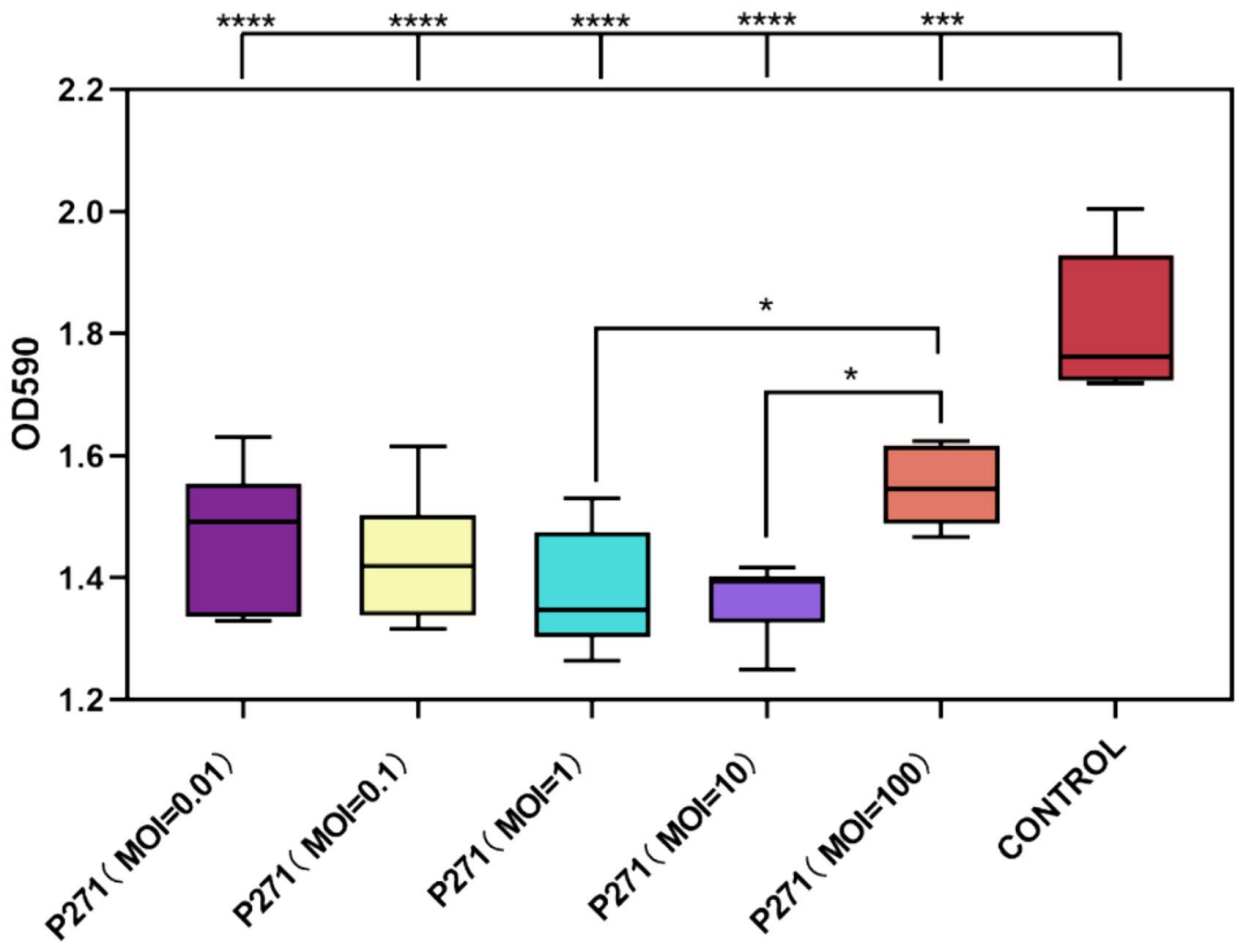
Efficacy of Phage P2-71 in Eradicating Mature Biofilms of *P. mirabilis* 37. This figure illustrates the comparative biofilm density following treatment with phage P2-71 at different MOIs. The control group represents the biofilm density of untreated *P. mirabilis* 37. Data are expressed as the mean optical density at 590 nm (OD_590_) from three independent experiments. Statistical significance was determined using One-Way ANOVA followed by Tukey’s multiple comparisons test, where * indicates *p* < 0.05, ***, p < 0.001, and ****, *p* < 0.0001.

As depicted in Figure 5, phages administered at varying MOI demonstrated effective eradication of *P. mirabilis* 37 biofilms after 24 h of co-culture with mature biofilms. The eradication rates ranged from 15 to 25%, with specific values of 19% at an MOI of 0.01, 21% at an MOI of 0.1, 24% at an MOI of 1, 25% at an MOI of 10, and 15% at an MOI of 100. Notably, phages at an MOI of 1 and 10 were more effective in reducing biofilm mass compared to the highest tested concentration (MOI of 100). However, these rates did not differ significantly from those observed at lower MOIs of 0.01 and 0.1. Statistical analysis confirmed the significance of these findings, with a *p*-value less than 0.001 for overall effectiveness and *p*-values less than 0.05 indicating differential effects across varying MOIs.

### Comprehensive evaluation of phage P2-71 efficacy in treating *Proteus mirabilis* infections

**Figure 6:**
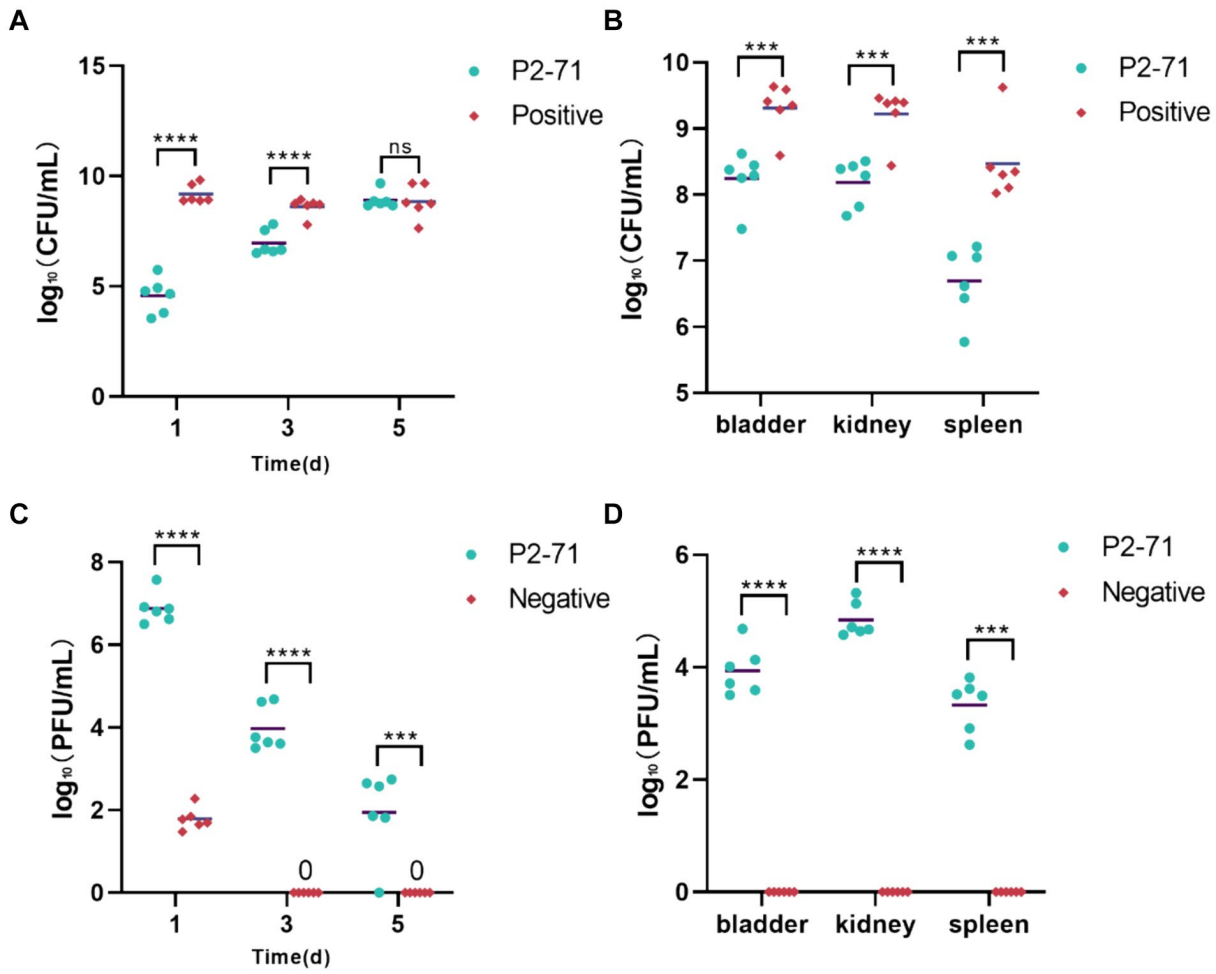
Treatment of UTI mice with phage P2-71. **(A)** Urinary *P. mirabilis* 37 loads determined by SS medium. **(B)** Internal organ *P. mirabilis* 37 loads determined by SS medium. **(C)** Phage titers of urine samples determined by the double plate method. **(D)** Phage titers of internal organ samples determined by the double plate method. “Positive” represents the group that received urinary injections of *P. mirabilis* 37. “Negative” represents the group that received urinary injections of phage P2-71. “P2-71” represents the treatment group that received both urinary injections of *P. mirabilis* 37 and subsequent urinary injections of phage P2-71. Statistical analysis was performed using the t-test followed by Tukey’s multiple comparison statistical test. ns indicates a non-significant difference (*p* > 0.05), *** indicates *p* < 0.001, and **** indicates *p* < 0.0001.

Initial treatment showed significant reductions in bacterial load in urine, with decreases of 4.602 log₁₀ CFU/mL on day 1 and 1.641 log₁₀ CFU/mL on day 3 (*p* < 0.0001). However, by day 5, the efficacy diminished as bacterial counts in both the treatment and positive control groups did not show significant differences (*p* > 0.05; Figure 6A). Additionally, phage P2-71 significantly reduced bacterial loads in the bladder, kidney, and spleen, with decreases of 1.169 log₁₀, 1.174 log₁₀, and 1.305 log₁₀ CFU/mL, respectively, all statistically significant (*p* < 0.001). This demonstrates the effectiveness of phages in eliminating bacterial loads from urine, urinary tract tissues and immune tissues (Figure 6B).

The phage titer in the urine of the treatment group was significantly higher than that of the negative group on the day 1(*p* < 0.0001). Over the following days, the phage titer in the treatment group exhibited a gradual decline, decreasing by 4.943 log₁₀ PFU/mL by day 5. In contrast, the phage titer in the negative group’s urine dropped to 0 PFU/mL by day 3. Further analysis revealed that on day 1, phage titers in the treatment group were substantially higher than in the negative group, indicating effective initial phage activity (*p* < 0.0001). Over the subsequent days, a marked decline in phage titer was observed in the treatment group, dropping by 4.943 log₁₀ PFU/mL by day 5. In contrast, phage levels in the negative group rapidly decreased to undetectable levels by day 3, suggesting a complete loss of phage activity (Figure 6C). Phage concentrations measured in the treatment group were 3.926 log₁₀, 4.632 log₁₀, and 3.211 log₁₀ PFU/mL across these organs. In contrast, no phages were detected in the negative control group, underscoring the targeted effectiveness of the phage treatment in reducing bacterial populations across multiple organ systems. This was further supported by significant differences in phage concentrations between the treatment and negative groups (*p* < 0.0001; Figure 6D).

### Assay of cytokines

**Figure 7:**
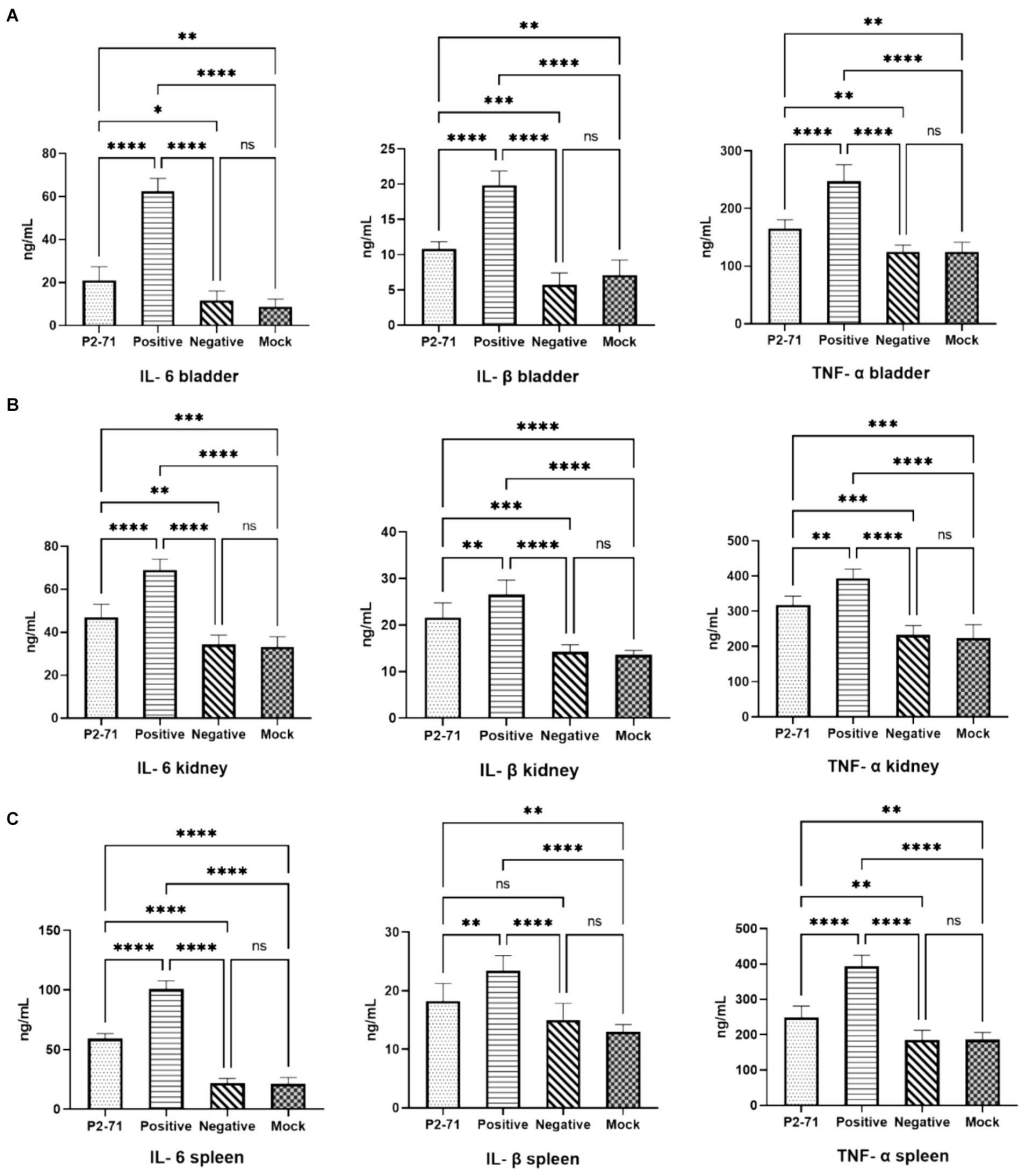
Concentrations of cytokines IL-6, IL-1β, and TNF-*α* detected by double-antibody sandwich assay. “Positive” represents the group injected with *P. mirabilis* 37, “Negative” represents the group injected with phage P2-71, “Mock” represents the blank group injected with PBS in the urinary tract, and “P2-71” represents the treatment group injected with phage P2-71 to treat *P. mirabilis* 37. **(A)** Bladder tissue: concentrations of IL-6, IL-1β, and TNF-α; **(B)** Kidney tissue: concentrations of IL-6, IL-1β, and TNF-α; **(C)** Spleen tissue: concentrations of IL-6, IL-1β, and TNF-α. The number of samples is 6 per group. Values represent the mean of each group, and error bars represent standard deviation. Statistical analysis was performed using a One-Way ANOVA test followed by Tukey’s multiple comparison test; ns indicates *p* > 0.05, * indicates *p* < 0.05, ** indicates *p* < 0.01, *** indicates *p* < 0.001, and **** indicates *p* < 0.0001.

In the bladder (Figure 7A), *P. mirabilis* infection significantly elevated the levels of inflammatory factors IL-6, IL-1β, and TNF-*α* (*p* < 0.0001). This elevation was significantly alleviated by treatment with phage P2-71 (*p* < 0.0001). Additionally, in the negative control group injected with phage alone, there was no significant increase in the levels of IL-6, IL-1β, and TNF-α compared to the blank control group (*p* > 0.05). In the kidney (Figure 7B), phage P2-71 treatment similarly reduced the *Proteus mirabilis*-induced increase in IL-6 (*p* < 0.0001), IL-1β (*p* < 0.01), and TNF-α (*p* < 0.01). A similar pattern was observed in the spleen (Figure 7C), an immune organ, where phage P2-71 significantly decreased the levels of IL-6 (*p* < 0.0001), IL-1β (*p* < 0.01), and TNF-α (*p* < 0.0001) compared to the infected positive control group.

### Histopathological analysis

**Figure 8:**
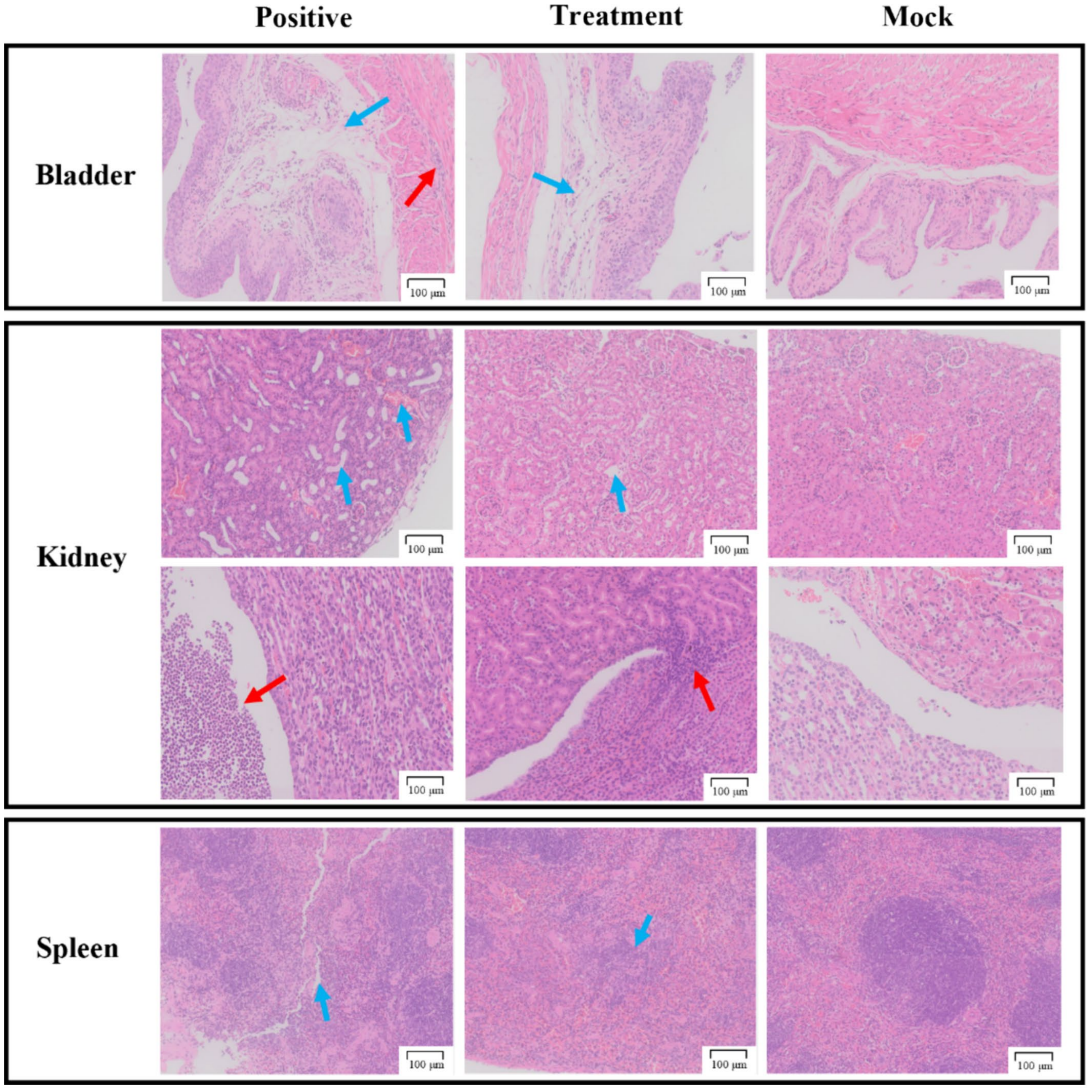
Pathological sections of tissue from the bladder, kidneys, and spleen. The first row of the kidney sections represents the cortical region, while the second row represents the medullary region. The “Positive group” consists of pathological tissue sections from urethra injected with *P. mirabilis* 37. The “Treatment group” includes pathological tissue sections from urethra injected with *P. mirabilis* 37 followed by phage P2-71. The “Simulated group” consists of pathological tissue sections from urethra injected with PBS. All scale bars represent 100 microns.

As shown in Figure 8, the bladder sections of the positive group displayed detachment of the bladder epithelium and infiltration of inflammatory cells in the muscularis propria. In contrast, the treatment group injected with phage P2-71 showed significant amelioration of *P. mirabilis*-induced epithelial detachment and inflammatory cell infiltration in the muscularis propria, compared to the positive group. The pathology sections of the mock group exhibited normal histology.

In the kidney sections, the positive group demonstrated tubular dilatation, congestion of the renal cortex, and substantial infiltration of inflammatory cells in the renal pelvis, compared to the mock group. The treatment group, however, showed only slight tubular dilatation and minimal inflammatory cell infiltration, indicating a protective effect of phage P2-71.

In the spleen sections, the positive group exhibited a reduction in red myeloid cells, protein exudation, and blurred boundaries between red and white myeloid cells compared to the mock group. The treatment group also showed a reduction in red myeloid cells but to a lesser extent than the positive group, suggesting a partial protective effect.

These findings indicate that phage P2-71 treatment effectively reduces pathological damage to urinary tract and immune tissues caused by *P. mirabilis* infection.

## Discussion

*Proteus mirabilis* is a gram-negative opportunistic pathogenic bacterium with flagella and hyphae that is widespread in natural and built environments, commonly found in the gastrointestinal tract of humans and animals. It can cause diseases of varying severity in a wide range of mammals, including humans, with urinary tract infections (UTIs) being one of the most notable conditions. Studies have indicated that UTIs are a significant reason for antimicrobial use in veterinary medicine, contributing to the development of antimicrobial resistance (15, 32). The overuse of antimicrobials, particularly third and fourth generation cephalosporins and fluoroquinolones, is common in companion animals with UTIs. This practice has allowed *P. mirabilis* to develop resistance to a wide range of antibiotics and acquire the ability to produce broad-spectrum *β*-lactamases (33), leading to serious public health concerns. In this context, phages are considered a promising alternative treatment to antibiotics due to their low side effects (34). In this context, phages are considered a promising alternative treatment to antibiotics due to their low side effects (35). In previous experiments, we take a strain of multidrug-resistant, strongly biofilm-forming *P. mirabilis* 37 isolated from canine feces. We used *P. mirabilis* isolated from feces as a bacterial pathogen for urinary tract infections because *P. mirabilis* is more abundant in the intestine. Furthermore, it has been shown that most urinary tract infections with *P. mirabilis* are caused by bacteria ascending from the intestine, and that *P. mirabilis* from the intestine has similar pathogenicity to urine isolates (4, 36). Not only that, the strains isolated from these feces demonstrated a high level of resistance phenotypes and resistance genes (9), which provides an even more challenging goal for our study to address a major veterinary public health problem such as antimicrobial resistance (AMR) and multi-drug resistance (MDR). We then isolated phage P2-71 using *P. mirabilis* as a host. Phage P2-71 belongs to the *Siphoviridae* family and demonstrates a notably brief latent period and a significant burst size per infected bacterium. Additionally, P2-71 exhibits strong thermal and pH stability. Genetic analyses revealed no virulence or resistance genes in P2-71, indicating its high therapeutic potential. These characteristics suggest that P2-71 is a viable candidate for phage therapy. Therefore, further *in vitro* and *in vivo* therapeutic trials with P2-71 are necessary to fully assess its potential efficacy.

First, we examined the susceptibility of *P. mirabilis* 37 planktonic bacteria to phage P2-71 in both PBS and urine environments. The experimental results showed that the phage reduced the amount of *P. mirabilis* in PBS by up to 1.537 log₁₀ CFU/mL (19% reduction compared to the control group). In the urine environment, it reduced the amount of *Proteus mirabilis* by up to 0.7009 log₁₀ CFU/mL (11% reduction compared to the control group). This bacterial reduction effect was relatively low compared to previous studies. For example, Maszewska et al. and Esmael et al. (29, 30) used OD values to quantify *P. mirabilis* and found that a single phage could reduce the bacterial load by more than 80%. Another study by Yazdi et al. (37) used viable counts and found that *P. mirabilis* was reduced by 5 log₁₀ CFU/mL within 3 h in the presence of phages. It is noteworthy that phages reduced *P. mirabilis* more effectively in PBS than in urine. This finding is consistent with a study using phage cocktails against *Enterobacter cloacae* (38), which demonstrated a reduction of 4 log₁₀ CFU/mL in PBS compared to only 2 log₁₀ CFU/mL in urine. The lower efficiency of phage inactivation in urine can be attributed to its lower pH, which affects phage activity despite their survival across different pH values (39). In the PBS environment, *Proteus mirabilis* began to gradually increase in the phage P2-71 treatment group from 6 h post-treatment. This phenomenon aligns with previous studies (29, 37, 38), suggesting that the bacteria may gradually develop resistance to the phage after prolonged exposure.

In biofilm assays, phage P2-71 demonstrated strong potential. When applied at different MOIs, phage P2-71 effectively inhibited biofilm formation by 34–49% after 24 h of co-culture with *P. mirabilis* 37. This inhibition range is moderate compared to previous studies. For instance, Carson et al. (40) reported a 10% reduction in *Proteus mirabilis* biofilm formation using phages. Esmael et al. (29) achieved reductions of 44.40 and 56.26% at phage concentrations of 8 log₁₀ PFU/mL and 9 log₁₀ PFU/mL, respectively. Additionally, Gomaa et al. (41) reduced *P. mirabilis* biofilms by more than 60% using a cocktail of three phages. Interestingly, our experimental results showed no significant difference in the ability of phages with different MOIs to inhibit biofilm formation. This suggests that phages with lower MOIs can be effectively used for biofilm prevention, potentially reducing manufacturing costs. In tests against mature biofilms of *Proteus mirabilis*, phages at different MOIs lysed 15–25% of the biofilm. This is lower than previously reported values. For example, Yazdi et al. (37) found that the isolated phage vB PmiS-TH lysed 89% of mature *P. mirabilis* biofilms at 8 h, though this percentage decreased to 70% after 24 h. Similarly, Maszewska et al. (30) reported a 50% lysis of mature biofilms using isolated phages. These findings align with the general observation that phages are more effective at preventing biofilm formation than lysing mature biofilms. This may be due to longer phage-bacteria interactions and the slower metabolic activity of bacteria in biofilms, which can slow phage proliferation and lead to the emergence of phage-resistant bacteria within the biofilm population (42). Our *in vitro* experiments have several limitations. For instance, we did not investigate the combined use of phage P2-71 with antibiotics. Numerous studies have demonstrated the synergistic effects of phage-antibiotic combinations on both planktonic bacteria and bacterial biofilms (43, 44). Examining these synergistic effects could provide valuable insights into the development of more effective therapeutic regimens. Additionally, this study did not explore the mechanisms by which *P. mirabilis* develops resistance to phage P2-71. Understanding these resistance mechanisms is crucial for optimizing phage therapy and preventing the emergence of phage-resistant bacterial strains. Future research should focus on identifying the genetic and phenotypic changes that confer phage resistance and exploring strategies to counteract these adaptations. Addressing these limitations could significantly enhance the efficacy and sustainability of phage therapy.

The experimental results of phage P2-71 treatment in a mouse model of urinary tract infection showed that the phage was effective in reducing the bacterial load in urine. Specifically, the phage reduced the bacterial load by 4.602 log₁₀ CFU/mL and 1.641 log₁₀ CFU/mL in urine collected on the first and third days, respectively. Previous studies using phages to treat *E. coli* urinary tract infections demonstrated that phages injected intraperitoneally or urethrally at a multiplicity of infection (MOI) of 10 were effective in clearing *E. coli* from urine within 24 h (~6 log₁₀ CFU/mL) (45, 46), an effect superior to that observed in our trial. Notably, by the fifth day, the bacterial load of *P. mirabilis* 37 in the urine was not significantly different between the treatment group and the control group, corresponding to a decrease in phage concentration in the treatment group. This observation aligns with our *in vitro* findings and is likely due to the emergence of phage-resistant bacteria. Despite this, phage P2-71 remained effective in reducing the bacterial load in the kidneys, bladder, and spleen. This is consistent with a previous study, which found that phage treatment reduced the bacterial load in the kidneys by 2 log₁₀ CFU/mL compared to the control group after 48 h (47). Another study demonstrated the potent ability of phages to eliminate bacteria, showing that a phage with an MOI of 10 completely cleared the bacterial load from the kidneys and bladder within 1 day in a chronic *E. coli* urinary tract infection model (46). Finally, elevated concentrations of TNF-*α*, IL-6, and IL-1β were detected in mice following the induction of UTIs, indicating an active inflammatory response. In contrast, this increase in cytokine levels was not observed in the negative control group injected only with phage, suggesting that the phage does not induce inflammation. Notably, the concentrations of TNF-α, IL-6, and IL-1β in urinary tract and immune tissues were significantly reduced following phage treatment. These cytokines are involved in cell proliferation, inflammation, and immune responses at both local and systemic levels and are commonly used to assess the degree of inflammation in UTIs in mice (48–50). The results demonstrated that phage treatment effectively attenuated both humoral and cellular immune responses. This finding is consistent with previous studies that reported a decrease in inflammatory cytokine levels following phage treatment (25, 26, 51). Additionally, pathological analyses revealed that the phage-treated group had relatively intact urinary tract and immune tissues with significantly reduced inflammatory cell infiltration. This suggests that phage therapy is effective in reducing the bacterial load of *P. mirabilis* in the bladder, allowing the host immune response to better defend against and clear the infection. These findings provide strong evidence supporting the application of phage therapy for the treatment of UTIs. However, there are some limitations to our animal testing. For example, phage therapy experiments were conducted in a controlled experimental environment, which may not fully replicate the complexity of natural infections encountered in different veterinary clinical scenarios. Additionally, the long-term efficacy of phage therapy and the potential for bacterial resistance to phages were not addressed in this study. Future research should focus on assessing the effectiveness of phage therapy in more complex and variable environments, as well as investigating the long-term efficacy and mechanisms of bacterial resistance.

In conclusion, our research provides a comprehensive evaluation of bacteriophage P2-71’s therapeutic potential against UTIs caused by MDR *P. mirabilis* 37. *In vitro* analyses demonstrated that phage P2-71 significantly reduced the bacterial population, effectively inhibited biofilm formation, and disrupted established biofilms. Correspondingly, *in vivo* studies revealed that phage P2-71 significantly decreased the bacterial burden in urinary tract tissues, reduced inflammation, and mitigated tissue damage in both urinary and immune organs. Collectively, these findings suggest that phage therapy offers a viable and innovative alternative to conventional antibiotics for managing infections caused by antibiotic-resistant bacteria. Given the rising concern of antimicrobial resistance, further research on phage P2-71 could pave the way for developing phage-based treatments as a crucial tool in veterinary infectious disease management.

## Data Availability

The raw data supporting the conclusions of this article will be made available by the authors, without undue reservation.
